# Non-Immersive Virtual Reality for Post-Stroke Upper Extremity Rehabilitation: A Small Cohort Randomized Trial

**DOI:** 10.3390/brainsci10090655

**Published:** 2020-09-21

**Authors:** Roxana Miclaus, Nadinne Roman, Silviu Caloian, Brindusa Mitoiu, Oana Suciu, Roxana Ramona Onofrei, Ecaterina Pavel, Andrea Neculau

**Affiliations:** 1 Faculty of Medicine, Transilvania University of Brasov, 500036 Brasov, Romania; roxicum@unitbv.ro (R.M.); caloian.silviu@unitbv.ro (S.C.); andrea.neculau@gmail.com (A.N.); 2 Rehabilitation Department, “Carol Davila” University of Medicine and Pharmacy, 0050474 Bucuresti, Romania; brindusailinca@yahoo.com; 3 Department of Rehabilitation, Physical Medicine and Rheumatology, “Victor Babeș” University of Medicine and Pharmacy in Timisoara, 300041 Timisoara, Romania; oanasuciu78@umft.ro (O.S.); onofrei.roxana@umft.ro (R.R.O.); 4 Faculty of Letters, Transilvania University of Brasov, 500030 Brasov, Romania; ecaterina.pavel@unitbv.ro

**Keywords:** virtual reality, post-stroke subacute, post-stroke chronic, neuroplasticity, upper-extremity rehabilitation, physiotherapy

## Abstract

Immersive and non-immersive virtual reality (NIVR) technology can supplement and improve standard physiotherapy and neurorehabilitation in post-stroke patients. We aimed to use MIRA software to investigate the efficiency of specific NIVR therapy as a standalone intervention, versus standardized physiotherapy for upper extremity rehabilitation in patients post-stroke. Fifty-five inpatients were randomized to control groups (applying standard physiotherapy and dexterity exercises) and experimental groups (applying NIVR and dexterity exercises). The two groups were subdivided into subacute (<six months post-stroke) and chronic (>six months to four years post-stroke survival patients). The following standardized tests were applied at baseline and after two weeks post-therapy: Fugl–Meyer Assessment for Upper Extremity (FMUE), the Modified Rankin Scale (MRS), Functional Independence Measure (FIM), Active Range of Motion (AROM), Manual Muscle Testing (MMT), Modified Ashworth Scale (MAS), and Functional Reach Test (FRT). The Kruskal–Wallis test was used to determine if there were significant differences between the groups, followed with pairwise comparisons. The Wilcoxon Signed-Rank test was used to determine the significance of pre to post-therapy changes. The Wilcoxon Signed-Rank test showed significant differences in all four groups regarding MMT, FMUE, and FIM assessments pre- and post-therapy, while for AROM, only experimental groups registered significant differences. Independent Kruskal–Wallis results showed that the subacute experimental group outcomes were statistically significant regarding the assessments, especially in comparison with the control groups. The results suggest that NIVR rehabilitation is efficient to be administered to post-stroke patients, and the study design can be used for a further trial, in the perspective that NIVR therapy can be more efficient than standard physiotherapy within the first six months post-stroke.

## 1. Introduction

Stroke Alliance for Europe states that “every 20 s, someone in Europe has a stroke”, while in the United States, “someone has a stroke every 40 s” a leading cause of significant long-term disabilities [1,2]. According to a European Union (EU) report, Romania has the lowest annual healthcare expenditure per capita (€1029 in 2015, compared to the EU average of €2884). The highest risk factors of a stroke are smoking and alcohol drinking, with males accounting for more than 50% of those impacted. Additionally, the level of education influences both lifestyle and life expectancy, with the Romanian life expectancy being among the lowest in the EU (75.3 years in Romania versus 80.9 years in the EU, in 2015). Moreover, there were 61,552 stroke cases in Romania in 2015 and forecasts state that this number will increase by 24% until 2035 [3,4].

Worldwide, the population faces high incidence rates of stroke and post-stroke sequelae with an increased need for neurorehabilitation services. In Europe, it is estimated that the number of annual stroke events will increase from 613,148 registered in 2015 to 819,771 in 2035, an increase of 34%. Considering that post-stroke survival rates have improved; estimations predict that the number of people living with strokes in Europe will grow from 3,718,785 in 2015 to 4,631,050 in 2035 [1].

Stroke complications can be long-lasting; thus, at 15-years post-stroke, two-thirds of survivors live with a disability, nearly two of five suffer from depression, and more than a quarter have cognitive impairment [5]. Post-stroke disability significantly contributes to the increasing use of long-term medical care resources, thus highlighting that efficient rehabilitation can cut costs in the healthcare system [6] whereas telerehabilitation is still in the early phase of utilization in developing countries.

Furthermore, international guidelines for stroke rehabilitation include physiotherapy techniques and methods for the recovery of the swallowing function and the urinary and bowel continence. These techniques and methods are also recommended for the improvement/prevention of shoulder pain, joint misalignments, and limb deviations caused by post-stroke spasticity, also used for secondary prevention of falling, as well as for enhancing the ability to perform self-care and daily living activities. Recovery from post-stroke impairments is facilitated, on the one hand, by increasing the motor function and, on the other hand, by improving the functionality of the limbs and body as a whole functional unit. In order to retrieve functional capacity, the existing guidelines recommend the use of intensive, repetitive training, improvement of functional mobility, use of orthoses, performing specific activities of daily living (ADLs) practiced repeatedly, progressive and bilateral training of the upper limb, the use of virtual reality and assisted robotic therapy, and the use of strength training exercises [7,8,9].

The use of virtual reality technology as an adjunct or substitute for traditional physiotherapy has been studied and proved to be effective in improving patients’ functional rehabilitation. However, as regards strokes, some systematic reviews suggest that virtual reality (VR) has not brought more benefits to patients compared to standard physiotherapy alone, while other research advocates for specific VR training as a therapy with a better outcome compared to conventional physiotherapy in the rehabilitation of stroke survivors [10,11,12,13,14].

Research on neuroplasticity and learning or relearning abilities shows that there are several principles of motor learning, including multisensory stimulation, explicit feedback, knowledge of results, and motor imagery. These principles, notably explicit feedback and multisensory stimulation, are found in the VR technology used for neuromotor rehabilitation. Accordingly, VR therapy becomes an alternative to classical physiotherapy, as it develops neuroplasticity. So, novel enriched environments are preferred in the context of current rehabilitation methods since guidelines do not provide an accurate record of evidence inferred from the specialized literature about motor skill learning. This evidence is essential in identifying practical methods and applications that could shape future approaches to neuromotor relearning. Furthermore, in animal research, it has been shown that aerobic exercise and environmental enrichment have pleiotropic actions that influence the occurrence of molecular changes associated with stroke and subsequent spontaneous recovery. These aspects may argue in favor of the efficient use of VR in motor and functional recovery after a stroke, by stimulating neuroplasticity [15,16].

Over the past ten years, research and literature reviews regarding the use of VR in post-stroke recovery have been homogeneous. Many approaches have focused on the use of VR as adjunct therapy alongside standard physiotherapy, and in some studies, non-dedicated VR technologies have been used, for medical purposes, in the motor rehabilitation of post-stroke patients [17,18]. Previous research on NIVR and immersive VR-based activities suggests that these interventions improve upper extremity rehabilitation after a stroke by providing motivating environments, stimulating extrinsic feedback, or simulating gameplay to facilitate recovery. Besides non-immersive VR therapy use in post-stroke patient’s rehabilitation, immersive VR therapy is used but requires more space and is more expensive, compared to NVIR. Robotic therapy is gaining more ground in neuro-motor rehabilitation, but the costs are very high, and in the case of exoskeletons, complex technology requires a long period of time for physiotherapists to acquire skills in the use of equipment. Currently, research has shown that VR positively influences the recovery of the upper extremity in post-stroke patients, as an adjunct therapy, by using dedicated and non-dedicated technologies [19,20]. The VR action on upper extremity post-stroke rehabilitation, using dedicated NVIR technology as a standalone therapy has not yet been determined at a staged level according to the post-stroke phases. The present study aims to investigate the efficiency of a dedicated NIVR system used in the rehabilitation of patients with subacute and chronic stroke, on upper extremity functionality and motor function. The research was done through specific VR training that incorporates real-time 3D motion capture and built-in visual feedback which provide functional exercises designed to train and regain the neuromotor functions of the upper extremity.

Our main goal was to evaluate the efficiency of the proposed protocol, by using staged, specific, and customized NIVR therapy on three levels of difficulty and by using specific exergames according to patient’s capacity, and adjusted by the level of difficulty, compared to standard physiotherapy. Besides, we were looking for differences in post-stroke clinical and functional status in the use of VR that improve or negatively influence the functional outcomes of the upper extremity when exposed to VR-based therapy compared to standard physiotherapy.

## 2. Materials and Methods

### 2.1. Participants

Initially, sixty-four patients were selected for the study, out of whom nine were excluded based on the exclusion criteria; fifty-five patients were admitted to the study undertaken in the Clinical Hospital of Psychiatry and Neurology in Brașov (Romania). They were all inpatients in the Neuro-rehabilitation Department. The inclusion criteria were:

(1) Stroke survivors after the acute phase, at least six weeks post-stroke; mild impairment (FIM ≥ 73, FMUE ≥ 13), minor cognitive impairment (Cognitive FIM ≥ 25);
(2) Stroke survivors within no more than four years after a stroke, at least 30-degree flexion and scapulohumeral abduction against gravity, and at least 30-degree elbow flexion against gravity.

The exclusion criteria were: severe cognitive impairments, global or transcortical sensory aphasia, anemia, atrial fibrillation, NYHA class IV heart failure, other dysfunctions in the upper extremity such as surgery, fractures, scapulohumeral periarthritis, or moderate-severe pain. These parameters of the physical and cognitive assessment may limit the possibility to apply the VR exergames with MIRA technology.

During the two-week follow-up period, three study participants, one from the chronic experimental group and two from the chronic control group, were removed from the research because they developed anemia and atrial fibrillation.

The study was conducted over nine months, from July 2019 to March 2020, and the patients were introduced to the therapy one by one (asynchronously). The Research and Ethics Committee approved the study of the Clinical Hospital of Psychiatry and Neurology in Brașov (no. 12534/18 July 2019). Informed consent was obtained from each patient after acknowledging his/her valuable participation in increasing the quality of life of future patients. Besides, they were all informed in advance about the possibility of drop out of the study at any moment, and that their participation in the research represents their consent to use and process personal data, according to the European legislation. The study was registered in clinicaltrials.gov, with no. NCT04436770.

### 2.2. Outcome Measures

Our research methodology consisted of the evaluation undertaken with four psychometric scales: Functional Independence Measure (FIM), Modified Rankin Scale (MRS), Modified Ashworth Scale (MAS), and Fugl Meyer Upper Extremity Assessment (FMUE). Manual Muscle Testing (MMT) and Active Range of Motion (AROM) were used to assess muscle strength and range of motion. The assessments were carried out by two experienced physiotherapists specially trained for this research and aimed to register data on stroke severity, activities of daily living, degree of spasticity, motor function and functionality, and the active range of motion. Their presentation and reliability are expressed below.

MRS was used to assess stroke severity. The reliability and validity of MRS have been assessed for test-retest reliability, and the weighted kappa statistic was excellent (kappa w = 0.95) [21]. Inter-rater reliability of the MRS was found to be excellent intraclass correlation coefficient (ICC) = 0.95 [22]. The concurrent validity criterion of the MRS was found excellently related to the Functional Independence Measure (FIM) (r = −0.089) while convergent/discriminant validity was 87% with the Barthel Index [23].

FIM was used for the assessment of ADLs. The psychometric properties of the FIM scale have been widely studied: (1) Internal Consistency proved to be excellent, with Cronbach’s alpha of 0.93 at admission and 0.95 at discharge. (2) Test-retest reliability was also excellent (ICC = 0.90 for Motor FIM and ICC = 0.80 for Cognitive FIM) [24]. (3) Intra-rater and inter-rater reliability showed excellent indices compared to the Barthel Index and among various raters. Content and criterion validity also proved that FIM is an adequate instrument to assess functional independence [24,25].

MAS was used to assess the degree of spasticity. Intra-rater and inter-rater reliability of MAS has proved to be an efficient tool for spasticity evaluation, especially for upper extremities [26]. Construct convergent validity was proven to be excellent compared to Fugl–Meyer Assessment (r = −0.94) [27].

The motor function and functionality were measured with a short Romanian version of FMUE [28] and MMT. FMUE reliability and validity were excellent for upper extremities (ICC = 0.95 for reliability and one factor for validity) [29]. Although controversial, MMT reliability and validity proved to be a useful instrument to assess muscle motor force [30].

AROM was also assessed for both control and experiment groups. Test–retest reliability scores of active ROM were excellent even among unskilled examiners [31]. The Functional Reach Test (FRT) was used to measure reaching distance [32].

### 2.3. Procedures

**Figure 1:**
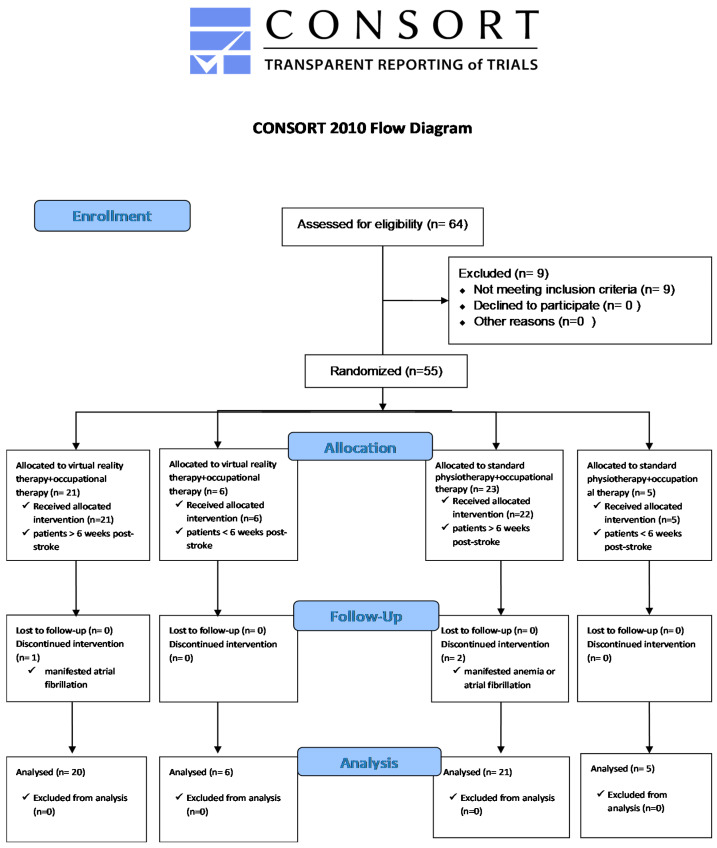
The CONSORT flow diagram.

Fifty-five participants were assigned to the experimental and control groups, using simple randomization. To avoid bias within our sample (*n* = 55), we used Graph Pad QuickCalcs to generate numbers that set patients into four groups. The allocation was performed using sealed opaque envelopes with the group name, which were placed in a plastic container in numerical order. The randomization procedure was performed by different individuals who were not involved in the intervention [33]. During the research, three participants were identified as having different health conditions which did not permit them to continue participating in the study. Therefore, fifty-two patients took part in the entire research program. The participants were divided into four groups: (1) Subacute Experimental (SE) enrolled patients less than six months since stroke (*n* = 6), and (2) Chronic Experimental (CE) enrolled patients more than six months since stroke (*n* = 20), both groups received VR therapy; (3) Subacute Control (SC) enrolled patients less than six months post-stroke (*n* = 5), and (4) Chronic Control (CC) enrolled patients more than six months since stroke (*n* = 21), both groups received conventional physiotherapy. All data regarding group allocation are presented in Figure 1, the CONSORT flow diagram. The duration of participation in the study for every patient was of 10 working days for two consecutive weeks. All four groups received a 60-min therapy session for upper extremities for ten days. The control groups benefited daily from a standard physiotherapy protocol of exercises such as self-passive mobilization, bilateral active mobilization, and active mobilization with resistance, task-specific functional exercises to increase the ability to perform ADLs, and dexterity exercises for the hand (occupational therapy exercises), for a total time of 60 min. The program of the experimental groups included 20 to 40 min of VR therapy (this duration was set according to the patient’s capacity) associated with dexterity exercises (occupational therapy exercises), so overall every patient performed one hour daily of upper extremity training. The protocol for occupational therapy exercises for the hand included the use of the Canadian plate, thick and thin grip training, lateral and palmar pinch, as well as wrist extensor strengthening tasks, for both groups.

### 2.4. Virtual Reality Software, Devices, and Exergames

The technology used consisted of a 55-inch TV screen, a computer running MIRA Rehab Limited, London, UK (a software for virtual reality therapy), and a Microsoft Kinect sensor that allows the detection of the human body, joints, and movements on all three axes. MIRA is a software (eHealth) telerehabilitation tool, which improves the effectiveness and convenience of physiotherapy for patients in recovery. The software uses the Kinect sensor to calibrate the patient’s position at the beginning of each VR session, or during exergames, if necessary. The technology used includes an evaluation tool for the AROM assessment, also performed through the Kinect sensor [34]. Through the MIRA program and the sensor, the patient receives feedback regarding the correctness of motion and posture during VR therapy sessions. During the research, the MIRA software was updated from version 2.2.3.0 (released on 16 July 2019) to version 2.2.5.8 (released on 19 December 2019).

The technology requires the presence of the therapists to assess the patient’s AROM, to determine the exergames tolerance level, and to establish the types of motion and exercise for the patient’s VR therapy session, according to the patient’s functional and motor capacity, at least at the beginning of the therapy (first two sessions). One example is the adjustment of the tolerance levels for motion from 0–100%. The lower the tolerance, the higher the software’s feedback on the correctness of movement, warning the patient that he/she is not performing the task accurately. The maximum level of tolerance used in our research was 20%, according to the manufacturer’s recommendations.

MIRA Rehab software has two categories of exergames based on types of motion, respectively a set of upper-limb analytical movements (see Supplementary Material), as well as a functional and complex set of exergames which involve muscle control, movement coordination, isometric contraction, and multiple directions of motion. The program features (through a bar on the left of the screen) types of motions that can be selected and (on the right of the screen) types of exergames, thus offering a multitude of possibilities for creating VR therapy programs. As for the functional exergames, the software features two types. One type of exergame can be used for coordination and movement control, for example, Firefly, Follow, Catch, Spaceship, and Move, which require the patient to follow an already designed complex path with three difficulty levels and another type of exergame with progressive difficulty sublevels. The software adjusts automatically the level of difficulty based on the patient’s assessed progress (from one session to another). MIRA VR therapy technology also assigns performance points for every exergame, according to the quality of the execution and the number of repetitions (within a set time), allowing patients to self-assess their progress and enabling them to receive proper feedback on their performance (quality of execution). The software provides a scoring system gathered at the end of the session, in addition to the feedback received during the execution of the exercises. Through this type of feedback, NVIR offers the patient the opportunity to see his progress through a computerized system, from the perspective of the quantitative evaluation of the progress, concretely counting on the results of therapy, and not just verbally.

For the study design, besides the assessment scales, to determine increased patient compliance in the use of VR, we considered two essential parameters, namely AROM and MMT. Firstly, we examined AROM in patients, both through VR technology and manually with the goniometer, where the muscle strength was below 3. In the cases of muscle strength above 3, AROM was measured in the antigravitational position and through MIRA technology. The tolerance level for AROM assessment was set to 20%. Within the first 10 patients, we assessed AROM with MIRA technology and the goniometer concurrently, to identify the level of tolerance needed for an accurate assessment and motion. All the directions of motion from the shoulder, elbow, and wrist were assessed. All reported AROM values are in degrees. For the personalized VR therapy, we adjusted the amplitudes of motion required for every of the chosen movement directions, according to the initial AROM assessment and to other assessment results included in the research (see Supplementary Material for additional information).

To encourage and motivate the patient to perform the tasks, we adjusted the tolerance level of the movement through the software at the beginning of the first two sessions.

The games used were customized according to the functional capacity of patients, divided into three groups according to their AROM and MMT capacity: limited, low, and high. For patients with low AROM and MMT capacity (less than 50 degrees and MMT between 2 and 3), we adjusted the virtual reality exergames by working bilaterally with both upper extremities (see Supplementary Material). If they had a limited AROM value, meaning that shoulder flexion and abduction were below 90 degrees (and MMT less than 3), the exergames were adjusted so that the subjects could do, as recreational activities, analytical movements of flexion, extension, abduction, and shoulder rotation, as well as analytical elbow movements, against gravity. For the patients with high shoulder mobility, with at least 90-degree flexion and abduction (and MMT at least 3), exercises involving complex movements were adapted. The multiple movements of the shoulder joint, involving the entire upper limb, were performed in the frontal-anterior, sagittal, and transverse plane. The elaborate motions were also adjusted to increase control and coordination so that movements could be performed diagonally, vertically, and randomly (see Supplementary Material).

Twenty percent of the exergames chosen (mostly, the same used for rehabilitation of movement coordination and kinesthetic proprioception) aimed at maintaining the upper extremity in isometric contraction during the exercise (1–3 min). For patients with MMT of at least 3, we increased the exergames difficulty level by attaching weights of 0.5–1 kg to the wrist, according to the patient’s strength. Throughout the virtual reality therapy sessions, the patients received specialized supervision and were initially guided verbally by a physiotherapist until they learned how to perform the exergames correctly. Patients were not seated unless they showed signs of imbalance (*n* = 7).

Due to the movement tolerance that can be adjusted in MIRA therapy, patients were warned to maintain the correct posture of the segment, for example, if they compensated with elbow flexion while they should have performed shoulder abduction. Also, patients received feedback at the end of every exercise, whereas in previous sessions/exergames, they had only received points.

Besides, all cooperation between patients and therapists was based on verbal and nonverbal communication. In the beginning, the therapy program was explained to patients in detail by therapists. The patients received clear learning-by-doing explanations and were also given a chance to exercise both specialized-assisted and self-automated NIVR therapies, the latter including no further human touch or correction from the therapists. In the first and second therapy sessions, patients needed verbal and physical guidance, but also an example of the movements they have to perform. Starting with the third session, most patients became familiar with the therapy used and needed verbal guidance, specifically only when the level of difficulty of the exergame changed. In the last sessions of VR therapy, patients no longer needed any guidance in exercise performance.

### 2.5. Statistical Analysis

Firstly, we investigated the assumption of normal data distribution, using Shapiro–Walk normality tests, which was violated, so the statistical analysis involved nonparametric tests. We used the independent Kruskal–Wallis test to determine if there were significant differences within our groups at the baseline. The Wilcoxon Signed-Rank test was used to determine the groups’ differences pre- and post-therapy, consecutively, independent Kruskal–Wallis with post hoc pairwise comparison was applied. We performed the statistical analysis using the IBM Statistical Package SPSS version 20.0 (IBM Corp. Released 2011. IBM SPSS Statistics for Windows, Version 20.0, Armonk, NY, USA). Multiple linear regressions with stepwise selection were applied to determine the best predictors and the relationships between the physiotherapy treatments and group characteristics and also to determine if any factor influenced or predicted the physiotherapy outcomes after a stroke. After the multiple linear regressions, we used the formula: dependent variable prediction = b0 + (b1 × b1 mean), where b0 = intercept and b1 = unstandardized coefficient, to determine the values of the variable’s prediction according to the outcome measures.

## 3. Results

Fifty-two participants completed the study, distributed by computer-generated randomization, out of whom six subjects were in the Subacute Experimental (SE) group, and 20 in the Chronic Experimental (CE) group, 5 in the Subacute Control (SC) group, and 21 subjects in the Chronic Control (CC) group. The characteristics of the patients from the investigated groups are presented in Table 1.

In Table 2, the results of the Wilcoxon Signed-Rank test regarding the pre- and post-therapy outcomes on the assessments performed are found. In both experimental groups, significant differences were obtained on AROM, MMT, FMUE, FIM, and FRT, while in control groups, significant differences were obtained in the chronic group (CG) on MMT, FMUE, and FIM assessments, while in the subacute control group (SC), significant differences were obtained on MMT, FMUE, and FIM assessments, but with values close to *p* = 0.05.

No significant differences were found regarding the baseline groups comparison for the outcome measures.

In Table 3, the results of the independent Kruskal–Wallis test are displayed, along with the values for group differences in the outcomes assessed. Excepting FRT, within all the assessments performed, differences were found, clarified by group pairwise comparison, with further understanding in the post hoc test, from Table 4.

By merging the data from Table 3 and Table 4, the results suggest that the subacute experimental group (SE) registered the most significant differences compared to control groups (subacute or chronic). As it regards AROM, MMT, FMUE, and FIM assessments, the subacute experimental group registered significant differences compared to the subacute control and chronic groups (SC and CC). Dealing with MAS values, both experimental groups recorded significantly lower values compared to the subacute experimental group. While within MRS assessment comparison, patients in the subacute experimental group had significantly lower values compared to patients in both chronic groups, either experimental or control.

As presented before in Section 2.5, multiple linear regression analysis was performed, and its results are listed in Table 5. Significant models were achieved by “VR time” variable, by “Post-Stroke Duration” (P-S.D), and by “dyslipidemia, diabetes, and Ischemic Coronary Disease (ICD)” as group characteristics. In Table 6, we listed the values of the predictors which influence post-stroke physical rehabilitation, according to the outcome measures. While “VR time” has a positive influence on upper extremity rehabilitation, “dyslipidemia, diabetes, and ICD”, and “long post-stroke duration” negatively influence upper extremity functional recovery. In rehabilitation, “long time” means more than one year since a stroke (in this research, from 1.1 to 4 years, as mentioned in Table 1).

No other correlations were found with the hemiparesis side, blood pressure, carotid artery atherosclerosis, age, or gender.

## 4. Discussion

The results of this study suggest that the use of NVIR-based therapy, as a standalone therapy, by a suitably-established protocol, gradually staged and adapted to the functional and neuromotor capacity of patients is efficient in the rehabilitation of the upper extremity in post-stroke patients. The use of VR as an adjunct to classical physiotherapy is considered to be efficient in patients with post-stroke sequelae, both for lower extremities, for the upper extremities, and also for balance [10]. Various research studies have used FMUE as a method for evaluating the upper extremity functionality and motor function and MMT for muscle strength assessment. Previous results suggest a significant difference between groups on the FMUE scale, while muscle strength reports have not shown significant differences pre- and post-therapy. In our study, the increase in muscle strength of the upper extremity may be explained by the addition of wrist weights during VR training and the study design [9,35].

The results of the Wilcoxon Signed-Rank test prove that VR therapy positively influenced upper extremity recovery for both groups receiving this type of therapy, for most of the assessments used, except MRS and MAS. The results are not surprising, considering that no significant differences were found in the control groups either; considering the duration of therapy and the fact that global scales like MRS and MAS are not very sensitive in a short time [36]. The results of the independent Kruskal–Wallis test shows statistically significant differences between the experimental and the control groups. The use of VR therapy can explain the results as a new enriched and interactive environment that influences neuroplasticity, especially regarding the subacute experimental group, whose participants are identified in the early post-stroke period, when the cerebral reorganization is manifesting at a maximum level [37].

Since no significant differences were found at the baseline for the four groups, we can emphasize that the results obtained can be based on the specific VR technology used for rehabilitation and the study design. Previous research showed that VR therapy could be more efficient in patients with a smaller degree of disability post-stroke. Our research suggests that this type of therapy has positive results even in cases of chronic stroke survivors when the therapy is adjusted, dedicated technology is used, the exercise program is staged, and used as a standalone form of therapy. Evidence pointed out that VR based treatments after a stroke could be 10% more efficient, with a higher activity rate than conventional therapy for severely impaired patients, suggesting that VR training facilitates more active training time. Although it might seem a short time of 20–40 min of VR therapy/day, the exergames are extremely soliciting, and patients execute only demanding motions during the VR training [38,39].

In the Cochrane review, devices dedicated to physical rehabilitation through VR and video-gaming devices were included in the study. Thus, due to the heterogeneity of the papers included in the research, the results suggested that VR therapy is not superior to classical physiotherapy. Another shortcoming of the papers analyzed is that AROM was not used as an objective method of assessment [12]. Thus, our research brings more knowledge regarding the personalized use of VR, using specific technology, compared to standard physiotherapy, as a standalone method and not as an adjunct therapy. Moreover, the patients’ compliance with the physical therapy program is another particular issue that may influence the results of using VR therapy as a method replacing conventional physiotherapy. The use of VR as a physical rehabilitation method represents an alternative form of therapy that attracts subjects to recreational activities, making them focus on the action of the game and not on the necessity of repetitiveness. VR technology is currently being explored for its potential benefits as a therapeutic intervention for relearning coordinated motion patterns. This technology lends the ability to create an environment in which the intensity of feedback and training can be systematically manipulated and improved to create the most appropriate paradigm of individualized motor learning. The use of VR also determines the development of motor skills and cognition, with a positive effect on the emotional state of patients with different types of neurological dysfunctions, increasing the compliance of the subjects with the treatment [40]. The patients move in front of a console and receive visual but also proprioceptive feedback about their movements through the sensor that projects on the screen the patient’s hand. Hence, they receive feedback on reaching the objectives, height, speed, and accuracy of movement. Thus, even if there is no immersion, the feedback is sensor-motor, where the patient’s movements in the real world are reproduced as the movements of an avatar on the distal segment in the virtual environment. Thus, VR allows more detailed sensor-motor feedback in the activation process, which can influence the recovery process [34].

Concerning motor learning, feedback refers to the information that the user receives as a result of a performed activity. By using VR systems, extrinsic or augmented feedback is exploited, which is related to the individual’s perception of the environment, primarily through the visual and auditory analyzer. Augmented feedback is relevant in terms of results knowledge and performance knowledge. Therefore, by using MIRA technology, the patient receives feedback on real-time performance by reaching the exergames objectives, but also by correcting the accuracy of the motion (when not performed correctly) or by the absence of motion correction warnings [41,42]. NIVR technology provides post-kinematic feedback on the pattern and the correctness of movement, which is essential for both learning and motor re-learning. Knowledge of the results appears after each exercise, but also after each training session, performing a comparative analysis with previous therapy sessions. Thus, through the NVIR technology used, the results of our research suggest that both augmented feedback mechanisms are involved, with positive influences in the recovery of the upper extremity in post-stroke patients.

The use of FIM as a measure of the results obtained in VR training confirms that VR therapy improves the patients’ both functional independence and upper limb function. The FIM outcomes in VR training arise even when compared to upper limb robotic therapy, as long as these new types of therapy are adapted and individualized to the patient’s ability [43].

The results of post hoc pairwise comparison for an independent Kruskal–Wallis test, suggest that the group of patients who received VR therapy and dexterity exercises, within less than six months post-stroke (SE) is the group with the relevant results and with visible differences from the other three groups, especially the control ones. VR therapy can induce neuroplastic changes even in patients in chronic stage post-stroke, by activating the ipsilesional premotor cortex and improving motor rehabilitation. As well as other environments that offer a blended form of standard physiotherapy, VR therapy, and occupational therapy which nowadays know an interdisciplinary approach from music, creative writing, dancing, visual arts, and bring unlimited research opportunities to the world of science and healthcare [44,45,46].

There is evidence that the use of video games has positive influences on both the cognitive component and learning and exercise, especially when using motion sensors that provide positive feedback. Moreover, recent research showed that there are visible changes in MRI after the application of other cognitive and neuro-muscular training modalities, such as video-gaming, and immersive and non-immersive virtual reality as a recovery therapy. Thus, positive influences are found both at the level of neuro-motor evaluation methods and also at the imaging level [47,48].

Most of the differences between the assessments result from the initial, and the final evaluations are found in the results of the linear regression which confirms that both the length of post-stroke time and the presence of diabetes negatively influence post-stroke recovery. Therefore, despite the patients’ heterogeneous profiles, ranging from subacute to chronic stroke phases, post-stroke recovery may be influenced by neuroplasticity and by the physiotherapy or rehabilitation environment as well. New studies show that even in the chronic post-stroke period, neuroplasticity is positively influenced by the use of VR therapy, with a positive impact not only on the motor function but also on the processes of the central nervous system, and with positive results on balance and posture [16,44,49,50].

The results of the linear regression applied to our variables show that time spent by patients in VR therapy is a factor that positively influences the increase in AROM, muscle strength, upper limb motor function (FMUE and MMT assessments), as well as the improvement of functional independence measured with FIM and MRS. Although the correlation coefficients in the linear regression have a weak effect size, in the case of MMT, FIM, and MRS, (<0.3) this may be influenced both by the number of participants in the study and especially by the number of days in which VR therapy was performed. That is why a proper assignation of an NVIR utilization protocol in a larger trial study is essential. An average period of 26.94 min of VR therapy increases AROM by 7.45 degrees of movement amplitude, muscular strength by 0.53 points, and functionality of the upper extremity (FMUE) by 9.68 points. The 26.94-min average use of VR therapy predicts an increase in FIM by 5.98 points and a decrease in MRS by 1.95 points in patients who are less than six months post-stroke and whose brain injuries are located on the right side. We also underline that patients’ recovery depends on the length of time since the stroke. Thus, as the post-stroke period increases, the FIM decreases by 2.54 points, and the MRS increases by 0.028 points. Issues related to increased disability one year after stroke have also been highlighted together with comorbidities such as cardiac diseases and other associated pathologies that negatively impact recovery [51]. In our study, dyslipidemia, coronary ischemic disease, and diabetes were the associated factors that negatively influenced the post-stroke physical recovery of the upper extremity. It is known that all these disorders belong to the associated risk factors of a stroke, still negatively influencing stroke recovery [52].

The strength of our research is that we conducted the first study of this kind in Romania, where NIVR is used in several centers in the country, less so for adults but preponderantly for children with neuromotor disorders. Another critical issue is represented by the stratified randomization, which allows for the obtaining of accurate data. In our view, VR technology should be developed into a form of virtual physical therapy or telerehabilitation, which is likely to gain more ground as SARS-CoV2 leads to an increased incidence of a stroke [53]. In such circumstances, after randomized controlled trial results, the possibilities of research will adapt this therapeutic program for post-stroke upper extremity rehabilitation to a self-controlled and adjusted version and to learning-by-doing outcomes. We also advocate that our research brings added value to telerehabilitation and e-health development in Romania. Since in the last sessions of VR therapy patients no longer needed guidance in performing the exercises, this is a premise for future research in the use of NVIR, after assisted training of patients as a post-stroke recovery therapy at home. A future NIVR therapy e-health program needs further design and research concerning individual and tele-assisted-use, including validated outcomes, as tools needed for maintaining patient’s outcomes after discharge or during hospital stay, and these outcomes should not be influenced by any lack of resources, irrespective of their nature (type, human, financial, or logistics).

### Limitations

A limitation of our research that require attention is related to the short duration of therapy of only ten days but this aspect is imposed by the policy of the national health system in Romania, and thus, we could not increase the duration of hospitalization. The medical unit in which the research was conducted has a neuro-rehabilitation department for patients in the subacute and chronic phase, in which patients are hospitalized for two weeks. One of the significant limitations of this study is related to the small number of participants in the subacute groups, either experimental or chronic. Thus, the obtained results cannot be extrapolated with certainty on the subacute post-stroke population, hence a future study with a larger number of participants in this category is necessary. A more extensive study would require at least 40 participants in each group to prove statistically significant changes in both impairment and upper limb activity of post-stroke patients. Based on the preliminary results, regarding the small number of participants in the category of subacute patients, the definitive utilization of non-immersive virtual reality therapy is debatable, to the detriment of conventional therapy for upper extremity rehabilitation. Based on the preliminary results, regarding the small number of participants in the category of subacute patients, it is debatable the definitive utilization of non-immersive virtual reality therapy, to the detriment of conventional therapy for upper extremity rehabilitation. Besides, another important issue that must be considered in a larger trial is related to the particularities of the post-stroke population, such as moderate impairment (e.g., FIM = 37–72), aphasia, a moderate or significant cognitive impairment which makes up a significant amount of the stroke population. The VR application is possible only in patients who meet the somatic and mental evaluation criteria already described in the inclusion criteria. By using a technology that requires attention, understanding, and feedback, our study does not address all patients who have suffered a stroke but provides significant results in accelerating recovery in patients with moderate deficits. In a future full randomized study, we suggest the possibility of a particular design, including an arm with patients experiencing aphasia and a more severe motor deficit. These criteria will lead to the applying of a particular set of exergames, starting from the most straightforward exercises and dedicating a longer VR therapy time, with more intense participation of the physiotherapist. However, the treatment with patients included in this arm is possible only for inpatients; it is impossible to use VR in the absence of a physiotherapist or at home. Nevertheless, given the progress of digitalization, e-health development and VR therapy use, new opportunities have arisen, and they can be explored through further research and in challenging or restrictive situations, like home rehabilitation, after discharge. Therefore, we emphasize the need, especially regarding chronic neurological patients, and the importance of a method to facilitate home VR therapy as a single or adjunct therapy to maintain the results obtained during hospitalization [54]. This outcome would decrease the costs for hospitalization and would reduce the gap between outcomes of inpatient rehabilitation and family, social, and professional reinstatement.

## 5. Conclusions

This study suggests the efficiency of specific NVIR use for upper extremity rehabilitation in post-stroke patients if special attention is paid to outcome measures, the length of VR therapy, and other particularities of the post-stroke population such as mild impairment, aphasia, or cognitive impairment. More extensive studies are needed to provide high-quality evidence for the specific effect of NVIR in the upper extremity and stroke rehabilitation. Our research was focused on studying physical rehabilitation of patients with post-stroke sequelae through NIVR, especially by using medical rehabilitation technology customized and adapted to the patients’ functional and motor capacities. The preliminary results suggest that in comparison with standard physiotherapy, NIVR has a favorable effect on upper extremity functional recovery, in patients with both recent and chronic strokes, but especially for patients less than six months post-stroke. Further research, with a larger effect size, is needed to identify the therapeutic window for VR therapy use as a tool for neuroplasticity enhancement and upper extremity rehabilitation after a stroke.

## Figures and Tables

**Table 1:** Patients’ characteristics.

Characteristic	Subacute Experimental (*n* = 6)	Chronic Experimental (*n* = 20)	Subacute Control (*n* = 5)	Chronic Control (*n* = 21)
Affected side (left/right)	3/3	10/10	2/3	5/16
Hemorrhagic/Ischemic stroke	2/4	5/15	1/4	7/14
Gender (male/female)	2/4	10/10	2/3	12/9
High blood pressure (yes/no)	6/0	16/4	4/1	16/5
Dyslipidemia (yes/no)	2/4	11/9	3/2	7/14
Ischemic coronary disease (yes/no)	2/4	12/8	0/5	9/12
Diabetes (yes/no)	1/5	5/15	0/5	1/20
**Age Groups**	**n/(%)**	**n/(%)**	**n/(%)**	**n/(%)**
41–50 years	2 (3.85%)	1 (1.92%)	3 (5.77%)	0 (0%)
51–60 years	1 (1.92%)	8 (15.38%)	0 (0%)	4 (7.69%)
61–70 years	3 (5.77%)	10 (19.23%)	0 (0%)	12 (23.08%)
71–80 years	0 (0%)	1 (1.92%)	2 (3.85%)	5 (9.62%)
**Post-stroke Duration**				
0–6 months	6 (11.54%)	0(0%)	5 (9.62%)	0 (0%)
7 months–1 year	(0%)	5 (9.62%)	(0%)	6 (11.54%)
1.1–2 years	(0%)	9(17.31%)	(0%)	7(13.46%)
2.1–4 years	(0%)	6 (11.54%)	(0%)	8 (15.38%)
**Mean (minutes)/SD VR Duration**	28.46/4.01	25.42/3.19	0/0	0/0
**Total Physiotherapy Duration (minutes)**	60	60	60	60

**Table 2:** Wilcoxon Signed-Rank test, pre- and post-therapy.

	SE Group (*n* = 6)			CE Group (*n* = 20)			SC Group (*n* = 6)			CC Group (*n* = 20)		
	Mean/SD	Mean Rank	*p*	Mean/SD	Mean Rank	*p*	Mean/SD	Mean Rank	*p*	Mean/SD	Mean Rank	*p*
AROM	9.41/2.86	3.50	0.028	9.00/4.48	10.50	<0.001	4.98/3.99	3.50	0.077	1.91/4.96	11.64	0.099
MMT	0.82/0.28	3.50	0.028	0.58/0.22	10.50	<0.001	0.36/0.29	3.00	0.041	0.44/0.14	11.00	<0.001
FMUE	12.66/2.42	3.50	0.027	8.45/3.61	10.50	<0.001	6.00/2.35	3.00	0.042	7.43/3.52	11.00	<0.001
FIM	10.00/2.44	3.50	0.026	3.65/4.33	9.50	<0.001	4.40/3.29	3.00	0.041	2.52/2.93	9	<0.001
FRT	5.16/2.78	3.50	0.027	4.88/4.54	9.00	<0.001	3.20/2.68	2.50	0.063	4.62/2.39	11.00	<0.001
MAS	0/0	0	1	0/0	0	1	0.40/0.55	0	1	0.05/0.22	1.50	0.157
MRS	−0.83/0.75	2	0.102	−0.10/0.31	2.5	0.056	−0.20/0.45	1	0.317	0/0	0	1

FMUE: Fugl–Meyer Assessment for Upper Extremity; MRS: Modified Rankin Scale, FIM: Functional Independence Measure; AROM: Active Range of Motion; MMT: Manual Muscle Testing; MAS: Modified Ashworth Scale; FRT: Functional Reach Test.

**Table 3:** Independent Kruskal–Wallis.

	Paired Differences						
	Mean	Std. Deviation	Minimum	Maximum	X^2^	df	*p*
AROM	5.79	5.57	−5.58	15.39	21.21	3	<0.001
MMT	0.52	0.24	0.18	1.13	13.19	3	<0.001
FMUE	8.28	3.71	2.00	17.00	11.49	3	0.009
FIM	4.00	4.12	0	15.00	13.14	3	0.004
FRT	4.64	3.39	0	19.00	1.40	3	0.704
MAS	0.05	0.23	0	1.00	12.16	3	0.007
MRS	−0.15	0.41	−2.00	0	18.36	3	<0.001

FMUE: Fugl–Meyer Assessment for Upper Extremity; MRS: Modified Rankin Scale, FIM: Functional Independence Measure; AROM: Active Range of Motion; MMT: Manual Muscle Testing; MAS: Modified Ashworth Scale; FRT: Functional Reach Test.

**Table 4:** Post hoc pairwise comparison for independent Kruskal–Wallis.

Outcome Measure	Pairwise Comparison	Mean Ranks	X^2^	Std. Error	*p*
AROM	SE > SC	36.50/22.00	20.08	4.73	<0.001
	SE > CC	36.50/15.79	20.71	7.01	0.019
MMT	SE > SC	41.50/12.40	29.10	9.17	0.009
	SE > CC	41.50/22.12	19.38	7.01	0.034
FMUE	SE > SC	44.58/17.00	27.58	9.13	0.015
	SE > CC	44.58/23.40	21.17	6.98	0.015
FIM	SE > SC	45.33/32.60	21.30	6.86	0.011
	SE > CC	45.33/22.02	23.31	6.82	0.004
MAS	SE < SC	25.00/35.40	−10.40	3.70	0.030
	CE < SC	25.00/35.40	−10.40	3.06	0.004
	CC < SC	26.24/35.40	9.16	3.04	0.016
MRS	SE < CE	12.42/27.45	−15.03	4.17	0.002
	SE < CC	12.42/30.00	−17.58	4.15	<0.001

SE: Subacute Experimental Group; CE: Chronic Experimental Group; SC: Subacute Control Group; CC: Chronic Control Group, FMUE: Fugl–Meyer Assessment for Upper Extremity; MRS: Modified Rankin Scale, FIM: Functional Independence Measure; AROM: Active Range of Motion; MMT: Manual Muscle Testing; MAS: Modified Ashworth Scale; FRT: Functional Reach Test.

**Table 5:** Multiple linear regression results.

Dependent Variable	Model	R^2^	*p* Change	Unstandardized Coefficients		Standard Coeff.	*p*	95.0% Confidence Interval for B	
				B	Std. Error	Beta		Lower Bound	Upper Bound
AROM	(Constant)	0.47	<0.001	4.08	0.90		<0.001	2.25	5.91
	VR Time			0.38	0.06	0.64	<0.001	0.25	0.51
	Dyslipidemia			−3.72	1.15	−0.33	0.002	−6.05	−1.39
MMT	(Constant)	0.16	<0.001	0.40	0.03		<0.001	0.32	0.48
	VR Time			0.01	0.00	0.55	<0.001	0.01	0.02
FMUE	(Constant)	0.31	0.001	8,17	0.68		<0.001	6.79	9.55
	VR Time			0.17	0.04	0.42	0.001	0.07	0.26
	ICD			−3.13	0.88	−0.42	0.001	−4.90	−1.35
FIM	(Constant)	0.29	0.011	4.51	1.07		<0.001	2.36	6.67
	VR Time			0.16	0.05	0.37	0.004	0.05	0.27
	P-S.D			−1.23	0.46	−0.32	0.011	−2.17	−0.30
MRS	(Constant)	0.23	0.029	−0.19	0.10		0.069	−0.41	0.01
	VR Time			−0.02	0.01	−0.44	0.001	−0.03	−0.01
	P-S.D			0.10	0.04	0.28	0.023	0.01	0.20
	Diabetes			0.33	0.14	0.27	0.029	0.03	0.62

FMUE: Fugl–Meyer Assessment for Upper Extremity; MRS: Modified Rankin Scale, FIM: Functional Independence Measure; AROM: Active Range of Motion; MMT: Manual Muscle Testing; MAS: Modified Ashworth Scale; P-S.D: Post-Stroke Duration; ICD: Ischemic Coronary Disease; VR: Virtual Reality.

**Table 6:** Multiple linear regression prediction scores.

Descriptive Statistics					
Linear Regression Models	*n*	Minimum	Maximum	Mean	Std. Deviation
Model 1 AROM	52	0.35	13.71	5.79	3.83
AROM-VR	52	4.08	13.70	7.44	3.56
AROM-Dyslipidemia	52	0.35	4.08	2.43	1.86
Model 1 MMT	52	0.40	0.76	0.52	0.13
Model 1 FMUE	52	5.03	12.47	8.28	2.07
FMUE-VR	52	8.17	12.47	9.67	1.59
FMUE-ICD	52	5.04	8.17	6.78	1.57
MODEL 1 FIM	52	−0.43	8.43	4.00	2.22
FIM-VR	52	4.52	8.69	5.97	1.54
FIM-P-S.D	52	−0.44	4.39	2.54	1.34
MODEL 1 MRS	52	−0.66	0.32	−0.15	0.23
MRS-VR	52	−5.20	−0.20	−1.94	1.85
MRS-P-S.D	52	−0.19	0.23	−0.02	0.11
MRS-Diabetes	52	−0.20	0.13	−0.15	0.11

FMUE: Fugl–Meyer Assessment for Upper Extremity; MRS: Modified Rankin Scale, FIM: Functional Independence Measure; AROM: Active Range of Motion; MMT: Manual Muscle Testing; P-S.D: Post-Stroke Duration; ICD: Ischemic Coronary Disease; VR: Virtual Reality.

## Supplementary Materials

The following are available online at https://www.mdpi.com/2076-3425/10/9/655/s1, Non-Immersive Virtual Reality for post-stroke upper extremity rehabilitation: a randomized pilot trial and study design.
