# Supplementary material for: Non-Immersive Virtual Reality for Post-Stroke Upper Extremity Rehabilitation: A Small Cohort Randomized Trial
Source: Brain Sci. 2020 Sep 21;10(9):655. doi: 10.3390/brainsci10090655 (PMC7563455; doi:10.3390/brainsci10090655)
Supplement: Supplementary file 1 [file brainsci-10-00655-s001.pdf]

Appendix 1: Non-Immersive Virtual Reality for post-stroke upper extremity rehabilitation: a randomized pilot trial and study design

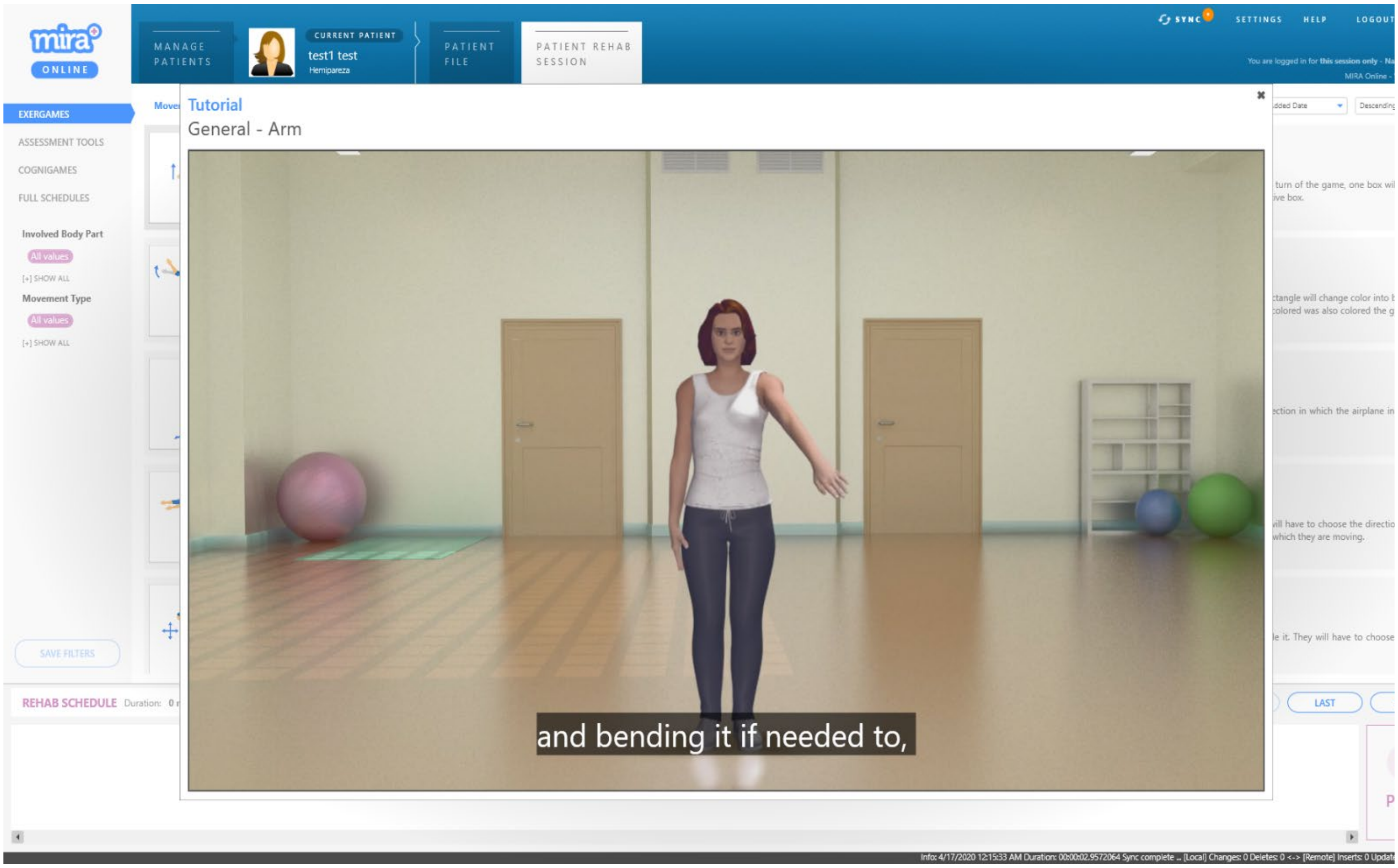

A. Exemplifying the patient interface – a tutorial presenting the motion required to the patient to perform, used at the beginning of the VR therapy or of every session

Appendix 1: Non-Immersive Virtual Reality for post-stroke upper extremity rehabilitation: a randomized pilot trial and study design

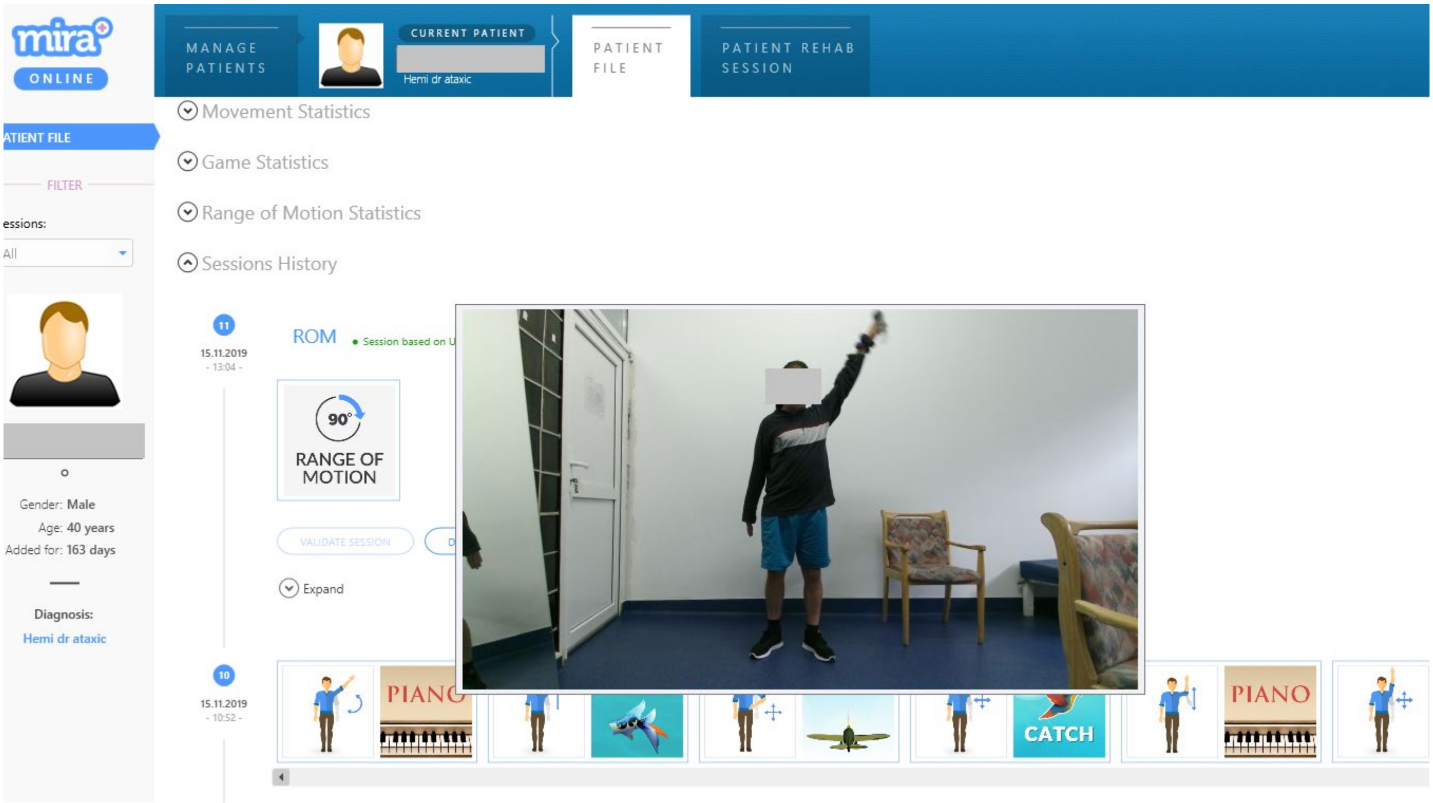

B. "Piano", an exergame for shoulder abduction performed with a weight on the wrist and a dumbbell in the hand

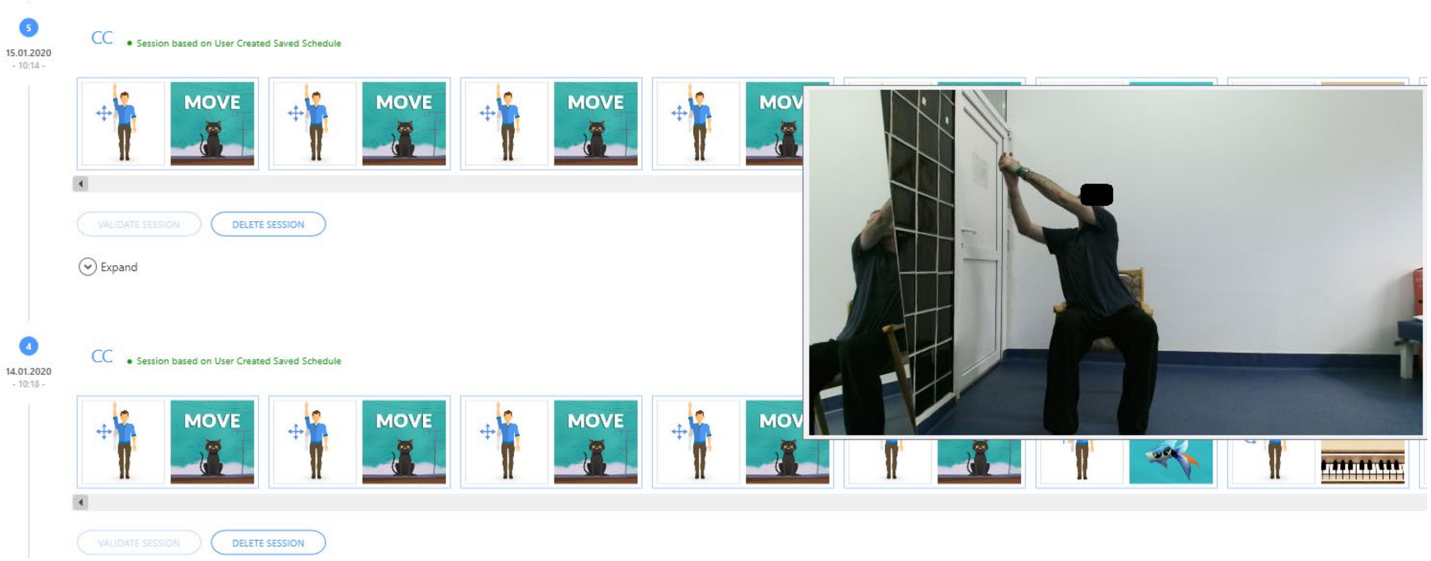

C. VR therapy for bilaterally training arms on different paths

Appendix 1: Non-Immersive Virtual Reality for post-stroke upper extremity rehabilitation: a randomized pilot trial and study design

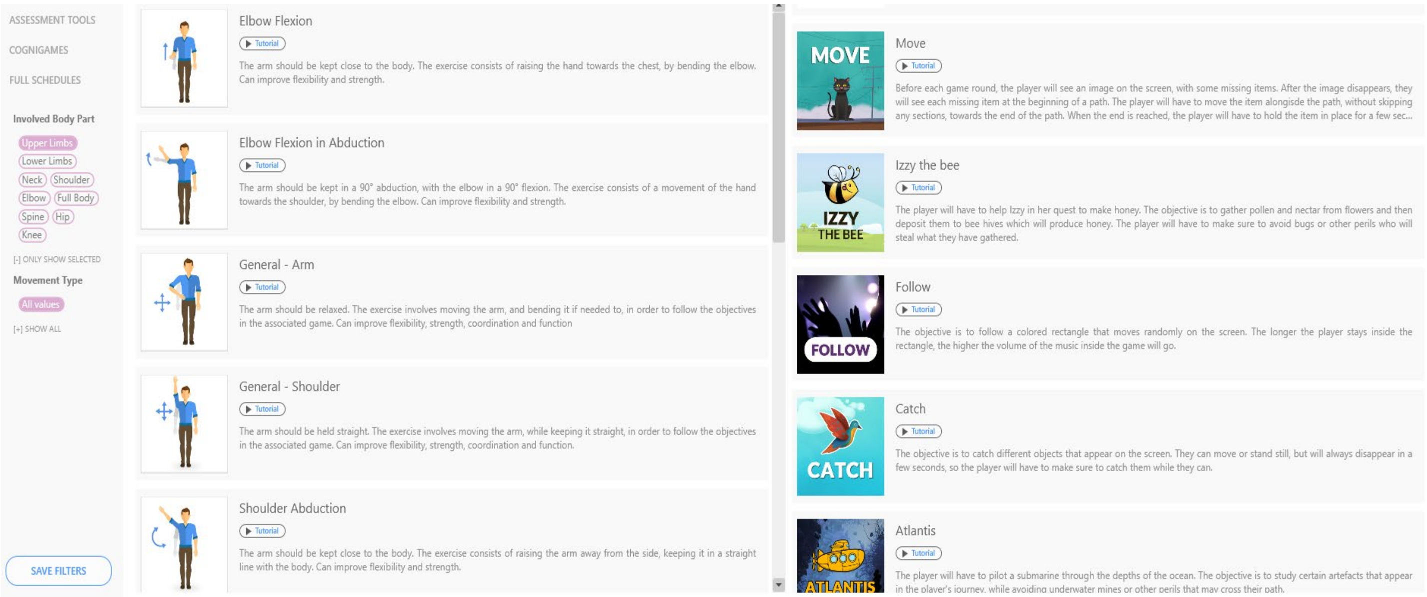

D. Selecting types of motions (in the left column) and types of the compatible exergames (in the right column)

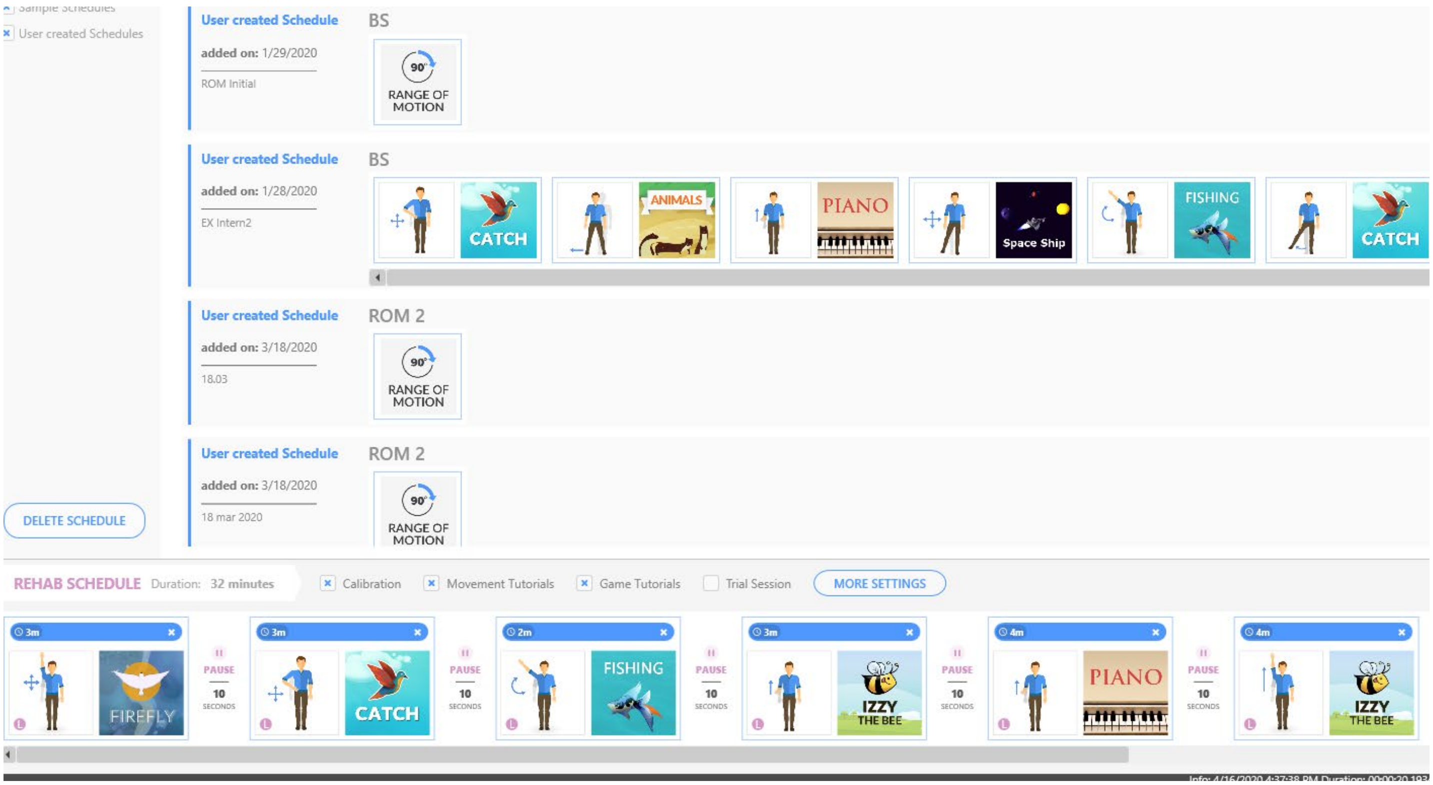

E. Types of exergames within the VR therapy

Appendix 1: Non-Immersive Virtual Reality for post-stroke upper extremity rehabilitation: a randomized pilot trial and study design

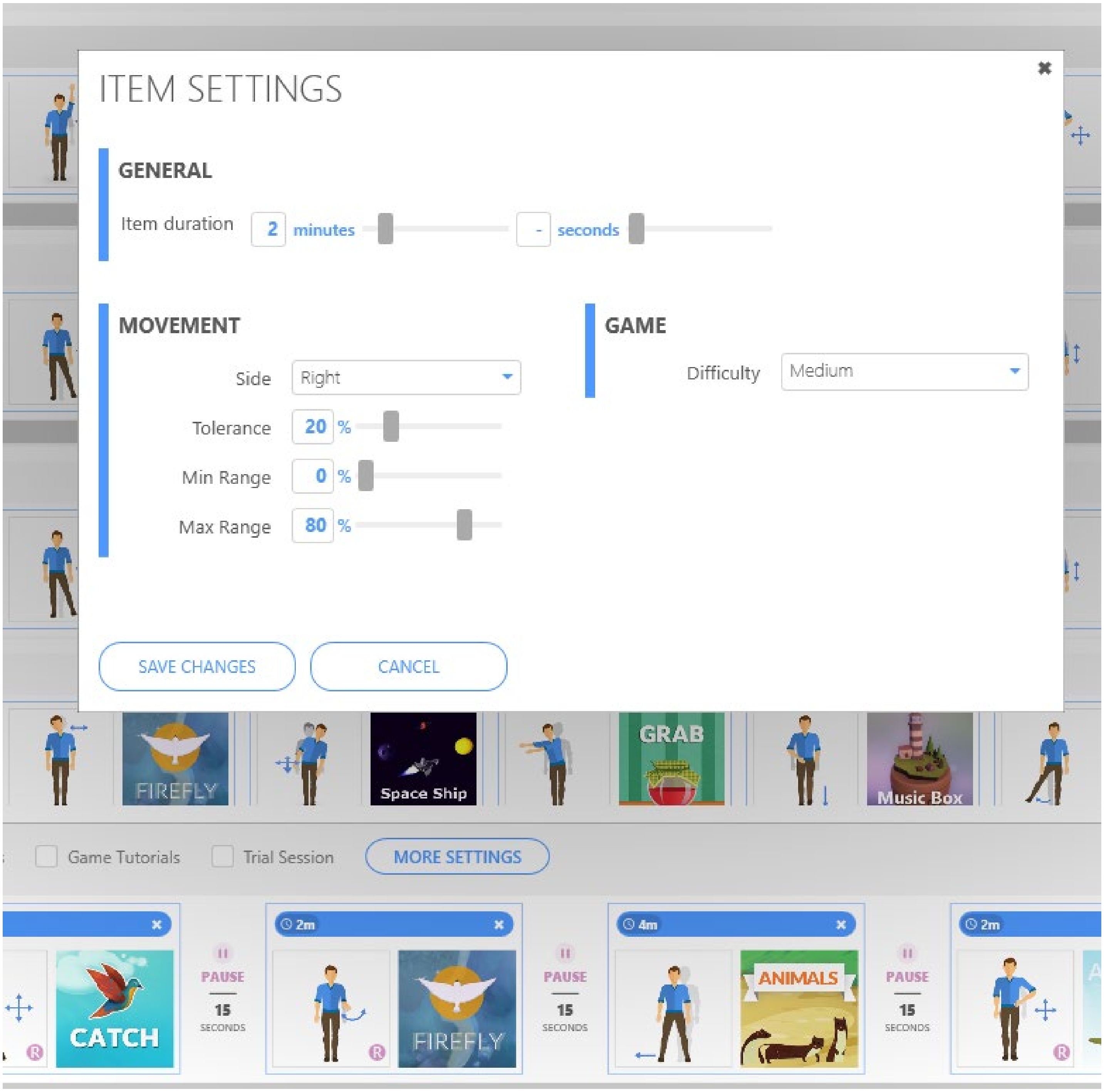

F. Types of MIRA adjustments: time, side of the limb (right, left or both, exergame-tailored), tolerance, motion amplitude (as min. and max. Range), and difficulty

Appendix 1: Non-Immersive Virtual Reality for post-stroke upper extremity rehabilitation: a randomized pilot trial and study design

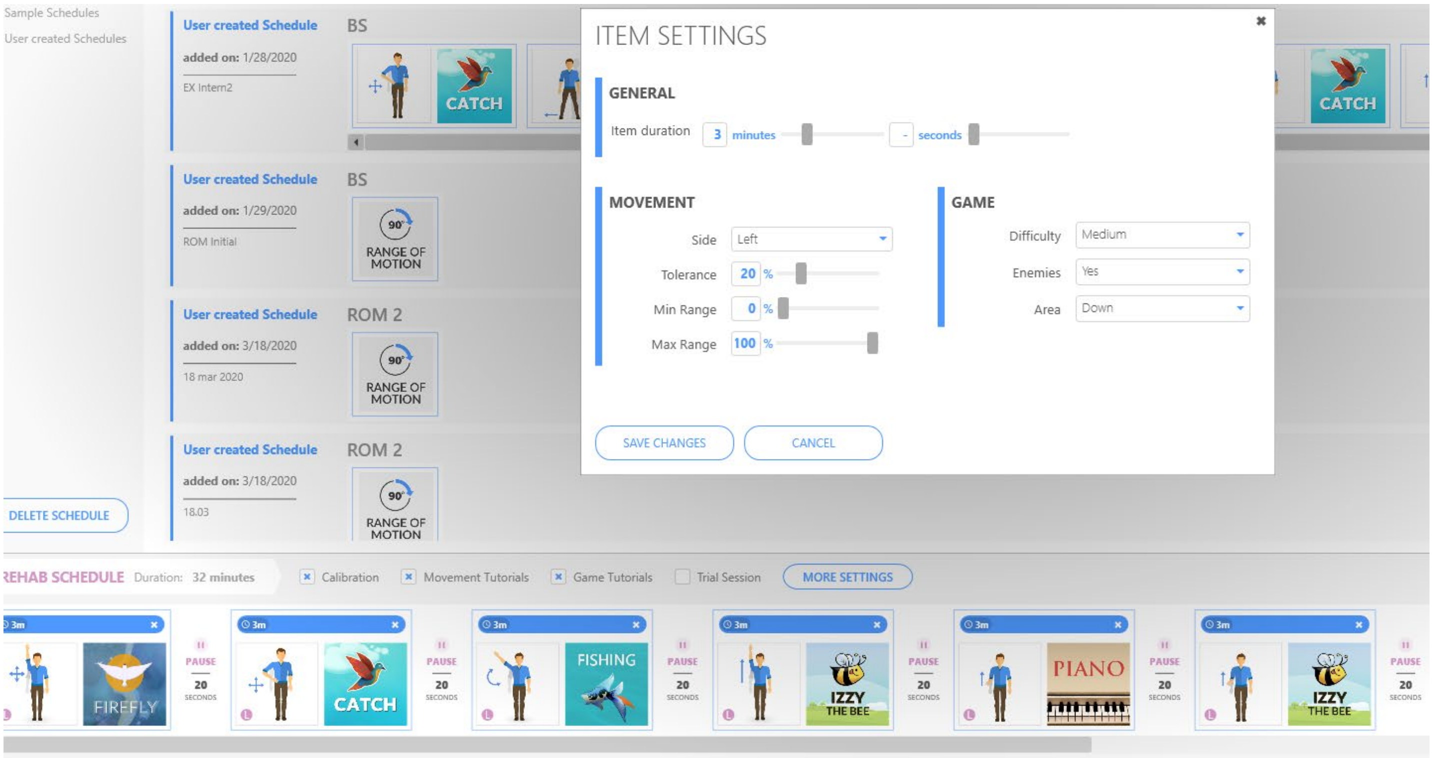

G. Adjustments within/Setting a specific exergame, "Izzy the Bee": based on the patient's needs, the therapist chooses obstacles and areas (up, down or both) for the patient to overcome when guiding a bee to collect pollen

Appendix 1: Non-Immersive Virtual Reality for post-stroke upper extremity rehabilitation: a randomized pilot trial and study design

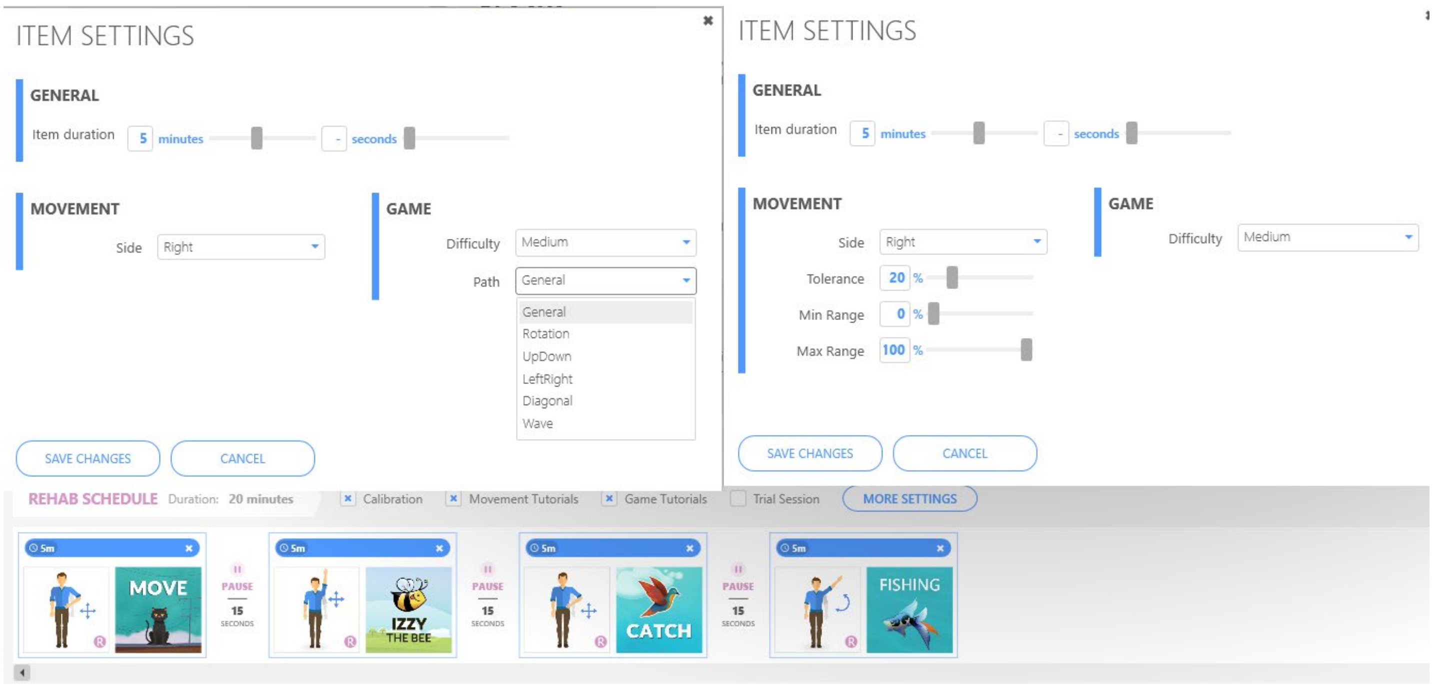

H. In the left column is showed the setting of a specific exergame- ”Move”. Based on the patient’s needs, the therapist can choose the game path for the patient (general, rotation, up and down, diagonal). Thereby, the patient simulates to ”grab an object” and move his upper extremity according to the chosen path. Control, coordination, and isometric contraction can be trained within this exergame. This exergame can be performed with one upper extremity or both.

In the right column, are presented the adjustments can be made for shoulder abduction using the ”Fishing” exergame. Within all analytical motions, the range of motion can be adjusted and should be set according to the patient's initial outcomes of the active range of motion assessment.
